# Facile Preparation of Durable Photothermal-Responsive PDMS-CB Surfaces for Droplet Manipulation

**DOI:** 10.3390/ma19101944

**Published:** 2026-05-09

**Authors:** Yan Hu, Guojian Yang, Xuyang Wu, Liming Liu, Kun Zhang

**Affiliations:** 1 School of Mechanical Engineering, Guangxi University, Nanning 530004, China; yh040900@163.com (Y.H.); guojyn@163.com (G.Y.); 19974073546@163.com (X.W.); 2 School of Mechanical and Electrical Engineering, Henan University of Technology, Zhengzhou 450001, China

**Keywords:** PDMS-CB composite coating, photothermal response, droplet manipulation, multi-modal

## Abstract

To address the issues of complex fabrication and poor mechanical stability associated with traditional light-controlled interfaces, we developed a highly durable photothermal surface based on a polydimethylsiloxane (PDMS)—carbon black (CB) composite using spin-coating and laser ablation techniques. Under 600 W/m^2^ illumination, the 3 wt% PDMS-CB surface achieves a rapid photothermal response, reaching 104.7 °C within 150 s. Driven by a localized 0.5 W near-infrared laser, a 10 μL droplet exhibits high-speed transport at 6.53 mm/s. Uniquely, the platform enables flexible, programmable multi-modal maneuvers, including dynamic obstacle avoidance, controlled merging, and induced splitting by presetting the laser spot trajectory. At the same time, the coating surface exhibits excellent durability when subjected to external mechanical damage. Notably, because the photothermal-active CB is uniformly embedded within the durable PDMS matrix rather than superficially coated, the surface maintains reliable actuation performance (4.17 mm/s) even after multiple cyclic actuation experiments. This study provides a simple, robust solution with potential for multifunctional integration for advanced non-contact microfluidic control and lab on chip applications.

## 1. Introduction

The precise manipulation of droplets has significant application value in the fields of microfluidic, biomedical detection, chemical micro-reactors and self-cleaning intelligent surface [1,2,3,4,5]. In recent years, to achieve efficient droplet transport and control, researchers have developed various active droplet propulsion technologies, including magnetic [6,7,8], electrical [9,10,11,12], acoustic [13,14,15,16], optical [17,18,19,20], and mechanical methods [21,22]. However, electronic control relies on dense electrode arrays and are limited in terms of openness and integration; magnetic control requires the use of magnetic materials, posing a risk of cross-contamination; acoustic control relies on piezoelectric materials, and high-frequency acoustic pressure can easily damage biomolecules; and mechanical force control carries a risk of surface structural fatigue. In contrast, optically controlled technology based on the photothermal effect has garnered significant attention due to its non-contact operation, high spatiotemporal resolution, and exceptional flexibility [23,24,25,26]. This technology does not require complex physical wiring or fluid additives; instead, it generates a significant temperature gradient by locally irradiating a photothermal surface with a specific light source. This temperature gradient directly disrupts the surface tension equilibrium at the droplet interface, which induce Marangoni convection [24,25,27,28] within the droplet inside the droplet. Then the droplets are driven to migrate continuously along the thermal gradient direction.

Although the photothermal induced droplet manipulation technology has made significant progress, the existing photothermal surfaces still face many limitations in practical applications. The preparation process of many photothermal responsive surfaces rely on expensive micro-nano fabrication equipment (such as: lithography systems, chemical vapor deposition (CVD) systems, or vacuum coating systems) and often involve cumbersome, multi-step chemical modification processes., which greatly limits the large-scale preparation of them [29,30]. Currently, the manipulation modes of some light-controlled surfaces are relatively limited, typically restricted to simple linear transport of droplets, making it difficult to flexibly achieve complex multimodal operations, such as: dynamic obstacle avoidance, on-demand splitting, and precise merging on a single platform [31,32]. Furthermore, the existing porous or micro-nano structured photothermal surfaces generally have the fatal weakness of poor mechanical stability. In the face of external mechanical friction, scratching or long-term recycling in practical applications, the surface microstructure is very easy to be damaged or peeled off, resulting in irreversible attenuation of hydrophobic properties and photothermal response ability, which seriously restricts the process of its application [31,33].

Our primary goal is to address the shortcomings of existing light-controlled droplet manipulation methods, such as: complex fabrication processes and poor mechanical stability, this study aims to develop a photothermal-responsive surface that is simple to fabricate, flexible to manipulate, and highly durable, thereby enabling efficient, multi-modal dynamic manipulation of droplets. Herein, we propose a durable photothermal surface based on a PDM-CB composite coating, fabricated via a facile combination of spin-coating and laser ablation. By embedding photothermal-active CB particles into the PDMS matrix and constructing hierarchical microstructures, the resulting surface achieves a synergy of rapid photothermal response and exceptional mechanical robustness. The PDMS-CB coating is unique in that it enables programmable multimodal control by adjusting the laser spot trajectory, including directed transport, dynamic obstacle avoidance, merging, splitting, and surface self-cleaning, offering a robust and flexible strategy for microfluidic applications.

## 2. Materials and Methods

### 2.1. Materials

Polydimethylsiloxane (PDMS, Sylgard 184) was obtained from Dow Corning (Midland, MI, USA), and silicone oil (viscosity: 100 cSt) was purchased from Shanghai Aladdin Reagent Co., Ltd. (Shanghai, China). Nano-carbon black (CB), used as the photothermal agent, was supplied by Guangdong Candlelight New Energy Technology Co., Ltd. (Dongguan, China). Specifically, the grade of the CB is VCX-72, featuring an average particle size of 30 nm and a specific surface area of 254 m^2^/g. The white pigment used in this study is a commercial water-soluble white dye, which was purchased from Anhui Zhanhao Bioengineering Co., Ltd. (Hefei, China). Methylene blue (Mb) was obtained from Bickman Biotechnology Co., Ltd. (Changsha, China). The glass microscope slide with dimensions of 25.4 mm × 76.2 mm was bought from Suzhou Rongyu Instrument Co., Ltd. (Suzhou, China). Anhydrous ethanol (99.7%) was provided by Shanghai McLean Biochemical Technology Co., Ltd. (Shanghai, China). All experimental water is deionized water made in the laboratory.

### 2.2. Preparation of the Photothermal Layer

**Figure 1:**
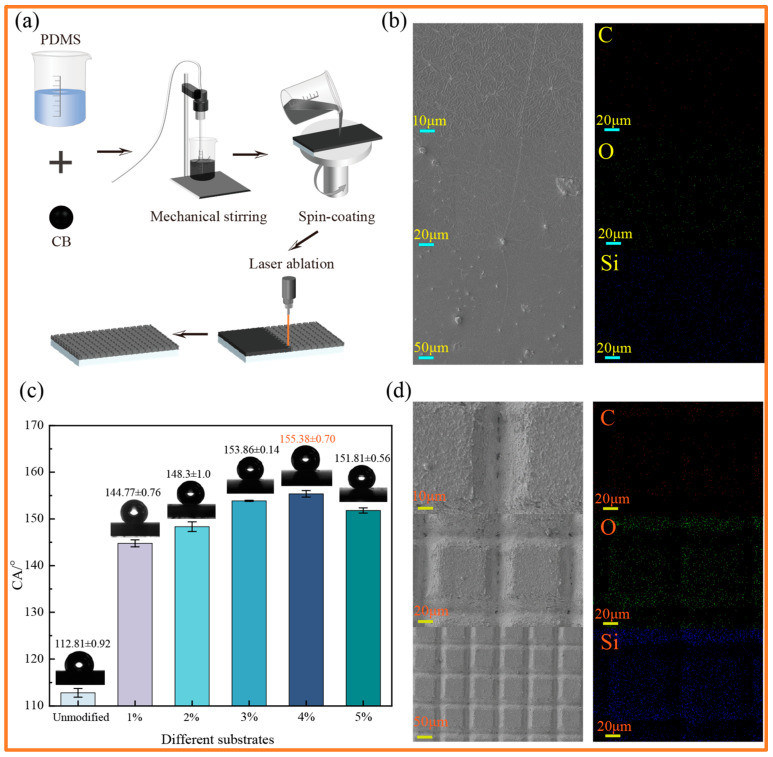
Preparation and Characterization of Photothermal Coatings: (**a**) Schematic of photothermal surface preparation; (**b**) SEM and EDS images of the PDMS-CB composite coating before laser processing; (**c**) CA images of the PDMS-CB composite coating before laser processing and after laser processing at different PDMS-CB content levels; (**d**) SEM and EDS images of the PDMS-CB composite coating after laser processing.

As shown in Figure 1a, to systematically investigate the effect of CB concentration on the subsequent photothermal and dynamic manipulation performance, a series of samples with CB mass fractions ranging from 1 wt% to 5 wt% were prepared. For each concentration, the PDMS prepolymer and CB particles were mechanically stirred at 500 rpm for 8 h to ensure thorough and uniform dispersion of the CB particles within the PDMS matrix. Subsequently, the curing agent was added at a weight ratio of 10:1 (silicone elastomer base to curing agent), and the mixture was stirred for an additional 15 min to obtain a homogeneous PDMS-CB solution. Next, the mixed solution was dripped onto a clean glass microscope slide. To ensure a uniform composite film, a three-stage spin-coating process was employed: the rotation speed was accelerated to 500 rpm within the first 30 s, maintained at a constant 500 rpm for 120 s, and finally decelerated to a complete stop within 30 s. The spin-coated substrates were then dried in a constant temperature oven at 75 °C for 4 h to fully cure the photothermal layer. Finally, the cured composite coatings were processed using laser ablation technology to construct a regular micro-array structure according to a preset program. The laser operational parameters were set as follows: a scanning speed of 10 mm/s, a laser power of 3 W, and a pulse repetition frequency of 1000 Hz. A grid pattern was uniformly ablated onto the PDMS-CB coating, with a spacing of 100 μm in both the x and y directions.

### 2.3. Photothermal Performance Test

To accurately evaluate the photothermal response, samples with varying CB contents (1–5 wt%) were placed on a thermally insulating white background to minimize thermal conduction to the bottom. The experiments were conducted in a windless indoor environment with a constant ambient temperature of 26 °C. An ULTRA-VITALUX sun lamp (OSRAM of Germany, made in Nové Zámky, Slovakia) was utilized as the illumination source. Prior to the tests, the incident illumination intensity was carefully calibrated to exactly 500 W/m^2^ using a solar power meter (Model: SM206-SOLAR (1999.9) (Xi’an Xinbao Scientific Instruments & Electronics Technology Co., Ltd., Xi’an, China). Meanwhile, the light source was positioned vertically above the samples to ensure uniform irradiation. The real-time surface temperature variations during the heating process were directly continuously recorded by a commercial infrared thermal imager (UTi320E, U-Lite Technology (China) Co., Ltd., Dongguan, China). The infrared thermal imager was set at an appropriate angle relative to the sample surface to monitor the temperature without obstructing the incident illumination path.

### 2.4. Droplet Dynamic Manipulation Performance Test

A super-slippery surface was prepared by evenly applying 0.2 g of silicone oil onto the patterned PDMS-CB coating. The sample was then left in a horizontal position for 10 min to ensure the formation of a stable and uniform lubricating layer. In dynamic manipulation tests, droplets of deionized water with predetermined volumes (5, 10, 15, 20, 25, and 30 μL) were placed on the prepared surface. A 808 nm continuous-wave laser was used as the driving light source. Directional transport was induced by precisely focusing the laser spot on the trailing edge of the water droplet. To evaluate manipulation performance, experiments were conducted within a laser power range of 0.1 to 1.0 W. Furthermore, by adjusting the laser beam path, complex multi-mode manipulations were performed, including obstacle avoidance, merging, splitting, and directed surface self-cleaning. The dynamic manipulation process was imaged using a camera system, and the captured video files were subsequently imported into Adobe Premiere Pro software (2022) for analysis. By tracking the displacement of the droplets over specific time intervals, the instantaneous and average velocities of the droplets were quantitatively calculated.

### 2.5. Photothermal Fatigue Stability Test

The photothermal fatigue stability of the coating under cyclic operational conditions was assessed by subjecting the 3 wt% CB sample to repeated laser illumination-cooling cycles. The ULTRA-VITALUX sun lamp was used as the light source. The incident irradiation intensity was strictly monitored and calibrated to exactly 600 W/m^2^ using the solar power meter. During each cycle, the surface was vertically irradiated for 10 min and subsequently allowed to naturally cool to the ambient room temperature (26 °C). This photothermal fatigue cycle was consecutively repeated 30 times. At the same time, to systematically monitor the degradation of surface wettability due to thermal fatigue, the static CA was measured after specific cyclic intervals. The CA measurements were performed using the standard sessile drop method with a contact angle system OCA 20 (Dataphysics, Filderstadt, Germany). For each test, the deionized water droplets with a precise volume of 5 μL were gently deposited onto the sample. To ensure statistical reliability, measurements were repeated at five randomly selected locations on the sample surface, and the data were averaged to calculate the mean CA and corresponding standard deviation.

### 2.6. Abrasion Test

The mechanical strength of the photothermal surface was evaluated via abrasion tests performed on samples containing 3 wt% CB. The coated side of the sample was placed face down on standard 1000-grit silicon carbide sandpaper. A vertical load of 100 g was applied to the back of the substrate using standard weights. The load was then moved back and forth horizontally for 20 cm at a constant speed of approximately 2 cm/s, defined as one complete abrasion cycle. This process was repeated continuously for a total of 125 cycles. The CA was measured after every 25 abrasion cycles to monitor the degradation of wettability. Additionally, to investigate changes in microstructure, a thin gold film was deposited on the surface after 25 abrasion cycles, and the surface morphology was observed using a scanning electron microscope (SEM, Merlin, Zeiss, Oberkochen, Germany).

### 2.7. Tape-Peeling Test

The adhesive tape peel test was used to evaluate the adhesion between the coating and the substrate. A 3M adhesive tape was firmly applied to the surface of the 3 wt% CB coating. To ensure uniform contact pressure and eliminate trapped air at the interface, a 1 kg roller was used to roll back and forth over the tape surface. Subsequently, the tape was peeled off at a 90° angle at a constant speed of 10 mm/s. Repeat this process and record the changes in the surface CA. After repeating this process 25 times, the structural integrity and morphological changes in the peeled surface were observed using the SEM.

### 2.8. Repeatability Test

Multiple droplet propulsion experiments were conducted on an optimized surface containing 3 wt% CB to investigate the stability of photothermal propulsion during long-term cycling and the durability of the lubricating layer. The propelled droplet consisted of deionized water, with its volume precisely controlled at 10 μL. Under irradiation from an 808 nm laser with a power of 0.5 W, the droplet continuously propelled along a predetermined straight-line trajectory for 68 mm, which was defined as one complete propulsion cycle. After each cycle, the surface was allowed to rest until the local temperature returned to room temperature (26 °C), and a new 10 μL droplet was applied. This photothermal propulsion process was repeated 30 times along the same trajectory.

## 3. Results and Discussion

### 3.1. Surface Morphology and Chemical Composition

As illustrated in Figure 1b,d, the surface of the initial PDMS-CB coating, prepared via spin coating, exhibits relative flatness and demonstrates an intrinsic hydrophobic state, with a CA of approximately 110°. However, the coating is impacted by the agglomeration of CB particles, resulting in an uneven distribution of surface elements. Following laser ablation processing, the high-energy beam induces localized gasification of the material. Observations from SEM images reveal the successful formation of a regular micron-scale array structure. Furthermore, the energy-dispersive X-ray spectroscopy (EDS) analysis indicates that laser processing effectively eliminated surface agglomerates. The EDS analysis indicates that the distribution of silicon (Si) and oxygen (O) elements on the roughened surface transforms from an initially non-uniform state to a highly uniform one, thereby providing a stable chemical foundation.

Figure 1c illustrates the influence of laser processing on surface wettability through contact angle (CA) measurements. The results showed that the smooth PDMS-CB coating, which had not undergone laser ablation, exhibited intrinsic hydrophobic properties, with an average contact angle of 112.81° ± 0.92°. After introducing microstructures via laser ablation, the CA value increased significantly, and the surface successfully transitioned to a superhydrophobic state (CA > 150°). Further statistical analysis revealed that as the carbon black (CB) mass fraction increased, the apparent contact angle exhibited a non-monotonic trend: the contact angle first increased gradually, reaching a maximum (155.38° ± 0.70°) at a CB content of 4 wt%, and then decreased slightly at higher CB concentrations.

The fundamental mechanism underlying this evolution in wettability lies in the synergistic interaction between laser-induced chemical homogenization and physically hierarchical roughness structures. From the chemical perspective, laser treatment uniformly re-exposes the stable Si and O atoms within the siloxane backbone, creating a low-surface-energy substrate. From the physical perspective, surface wettability is highly sensitive to the hierarchical synergy between laser ablation microstructures and CB-induced nanoscale roughness. The regular laser-ablated microarrays align closely with the Cassie-Baxter wetting model: the micro-nano structures effectively trap air to form an air layer, thereby significantly reducing the solid–liquid contact area and supporting the droplets. Meanwhile, When the CB content is below 4%, the density of nanoparticles is insufficient to form the nanoscale protrusions required to support a stable gas cushion, thereby failing to maintain optimal superhydrophobicity. Conversely, once the CB exceeds 4%, an excess of nanoparticles induces severe physical agglomeration. This agglomeration disrupts the spatial regularity and uniformity of the laser-patterned microstructure, creating structural heterogeneity that leads to drastic fluctuations in local capillary forces. Simultaneously, this structural disruption causes droplets to penetrate the microchannels, triggering a localized transition from the Cassie-Baxter state to the Wenzel wetting state, ultimately resulting in the observed decrease in CA [34,35].

### 3.2. Photothermal Response Performance

**Figure 2:**
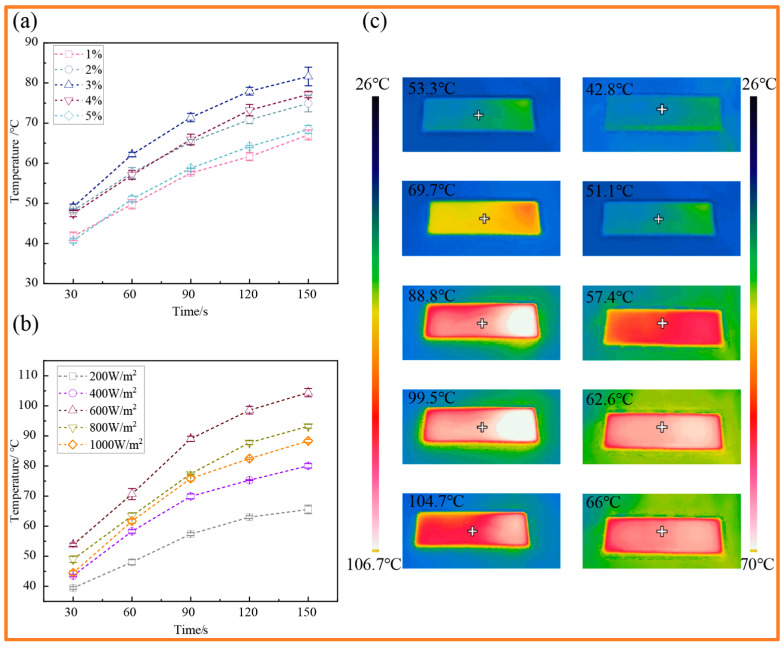
Photothermal response performance test: (**a**) Temperature rise curve under different concentrations of PDMS-CB content. (**b**) The temperature rise curve (CB content is 3%) under different light intensities. (**c**) Real image of infrared thermal imaging during temperature rise: left side shows 3% CB content at 600 W/m^2^ light intensity; right side shows 1% CB content at 500 W/m^2^ light intensity.

The photothermal response of the coating under 500 W/m^2^ illumination initially increases and subsequently diminishes with increasing CB mass fraction, achieving the optimal temperature rise effect at a 3% addition level (Figure 2a). Figure 2b demonstrates that, with a fixed CB content of 3%, the photothermal response of the coating significantly enhances with increasing incident light intensity, exhibiting the best heating performance under 600 W/m^2^ illumination. At this juncture, the conversion efficiency from light energy to thermal energy reaches its peak, ensuring not only energy-efficient heat input but also preventing light and heat saturation as well as severe heat convection dissipation losses caused by excessive light intensity. During the dynamic heating process, the coating efficiently absorbs light within the first 90 s, leading to a rapid increase in surface temperature; from 90 to 150 s, as the temperature differential between the surface and the environment widens, heat loss escalates, and the heating rate gradually decelerates until steady-state thermal equilibrium is attained. The photothermal response efficiency is significantly related to the concentration of CB, and is greatly affected by its spatial distribution in the composite coating. When the mass fraction of CB is below 3%, the CB particles are uniformly dispersed in the PDMS matrix, thereby maximizing the effective specific surface area available for light absorption and energy conversion. However, once the CB concentration exceeds 3%, physical agglomeration of the CB particles inevitably occurs, leading to a decrease in photothermal response efficiency. On the other hand, these micron-scale aggregates introduce substantial interfacial thermal resistance at the CB-PDMS interface. This thermal resistance hinders heat conduction from the CB to the PDMS matrix, leading to localized heat accumulation and dissipation into the environment, which results in a decrease in photothermal efficiency [36,37,38,39,40]. As depicted in Figure 2c, the coating surfaces under conditions of 3% CB at 600 W/m^2^ and 1% CB at 500 W/m^2^ both exhibit a continuous and uniform thermal field, devoid of localized hot spots, confirming the homogeneous distribution of CB. The primary disparities between the two conditions lie in the heating rate and steady-state temperature: the former rapidly heats up and achieves a high-temperature steady state of 104.7 °C across a large area, whereas the latter exhibits a sluggish thermal response with a significantly reduced steady-state temperature of 66 °C.

### 3.3. Dynamic Droplet Manipulation Capabilities

**Figure 3:**
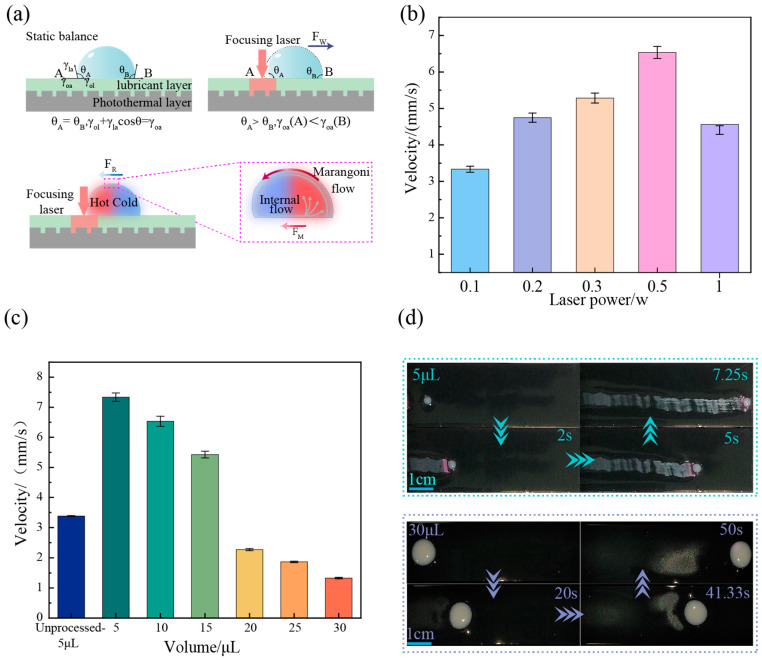
Dynamic manipulation of droplets: (**a**) Schematic illustration of the principle for droplet manipulation on a superhydrophobic surface based on photothermal response. (**b**) Relationship between laser power and droplet velocity. (**c**) Relationship between droplet volume and velocity. (**d**) Physical photographs capturing the movement process of droplets with volumes of 5 μL and 30 μL.

When the interior and interface of a droplet are subjected to regulation by an asymmetric thermal field, the Marangoni force ($F_{M}$) and wetting gradient force ($F_{W}$) are generated, driving the droplet to move in a specific direction. Figure 3a illustrates the schematic diagram of the principle for manipulating droplets on an optically controlled super-slippery surface based on photothermal response. This functional surface primarily consists of a photothermal layer and a lubricating layer at the bottom, which serves to reduce the resistance encountered by the droplet during sliding. In the absence of external light excitation, the droplet remains in thermodynamic equilibrium on the super-slippery surface, with equal contact angles on both sides (*θ_A_* = *θ_B_*). The relationship between interfacial tension and contact angle can be described by Young’s equation [41]:

(1) $$\gamma_{oa}-\gamma_{ol}=\gamma_{la}cos\theta$$

where $\gamma_{oa}$ represents the oil–gas interfacial tension, $\gamma_{ol}$ denotes the oil–liquid interfacial tension, and $\gamma_{la}$ signifies the liquid–gas interfacial tension. When a laser with a specific power asymmetrically irradiates side A, the photothermal layer at the bottom absorbs the light energy and converts it into heat energy, establishing a significant temperature gradient within and around the droplet. Due to the presence of a local heat source, the liquid surface tension in zone A (the high-temperature side) is lower than that in zone B (the low-temperature side). According to the Marangoni effect, the fluid tends to flow spontaneously from regions of low surface tension to regions of high surface tension. This thermocapillary effect induces a distinct directional flow field within the droplet, resulting in a tangential stress F_M_ directed from the hot end to the cold end, with the expression given by [42,43,44,45,46]:

(2) $$F_{M}≅\pi R^{2}\frac{d\gamma_{la}}{dT}\frac{dT}{dx}$$

Here, R is the radius of the droplet, x represents the direction of droplet movement, $\frac{d\gamma_{la}}{dT}$ is the change in liquid–gas interfacial tension with temperature, and $\frac{dT}{dx}$ is the rate of temperature change along the direction of droplet movement. Simultaneously, the rapid increase in local temperature not only induces an internal flow field but also directly alters the macroscopic stress state at the droplet interface. The significant temperature rise leads to a decrease in oil–gas interfacial tension $\gamma_{oa}$(A) and an increase in contact angle *θ_A_* in the illuminated area, disrupting the equilibrium state of the contact angles *θ_A_* and *θ_B_* on both sides of the droplet. This asymmetric change in contact angles disrupts the original force balance of the droplet, resulting in an $F_{W}$ that is also directed away from the light source, with its magnitude approximately expressed as [47,48]:

(3) $$F_{W}≅2R(cos\theta_{B}-{cos\theta }_{A})$$

The direction of resistance ($F_{R}$) during droplet movement is generally opposite to the movement trend of the droplet. It is composed of the wetting ridge formed at the bottom of the droplet on the lubricating surface and its internal viscous force, which is primarily proportional to the following parameters [49]:

(4) $$F_{R}\propto (\mu_{l}+\mu_{o})\pi Rv$$

where $\mu_{l}$ and $\mu_{o}$ are the viscosities of the droplet and the silicone oil, respectively, and v is the velocity of the droplet. When *F_M_* + *F_W_* > *F_R_*, the droplet begins to accelerate and move towards side B. While *F_M_* + *F_W_* = *F_R_*, the droplet moves towards zone B at a constant speed.

Based on the aforementioned physical mechanism, this paper further quantitatively investigates the influence of manipulation parameters on droplet motion performance. For a fixed droplet volume of 10 μL, the propulsion velocity initially increases and subsequently decreases as laser power rises, peaking at 0.5 W with a velocity of 6.53 mm/s, which was illustrated in Figure 3b. During the initial stage, the increase in power enhances local photothermal conversion and establishes a steeper temperature gradient, significantly improving the driving force. However, when the laser power exceeds 0.5 W, excessive heat input leads to local thermal saturation and excessive thermal dissipation, weakening the effective surface tension gradient and reducing the slip velocity. Figure 3c reveals the synergistic effect of surface microstructure and droplet volume on slip velocity. At a laser power of 0.5 W, the velocity of a 5 μL droplet can reach 7.19 mm/s, while the movement velocity of a 5 μL droplet on an unprocessed photothermal surface is 3.38 mm/s, and the velocity of a 30 μL droplet is 1.33 mm/s. Compared to the original coating without laser processing (which lacks microstructure and unable to effectively retain the lubrication layer, resulting in poor friction reduction during movement), the laser-processed surface significantly reduces contact angle hysteresis by introducing microtextures, thereby significantly improving the slip velocity of droplets under the same conditions. Simultaneously, on the processed surface, the droplet velocity and volume exhibit a negative correlation trend, attributed to the significant increase in mass and three-phase contact line of large-volume droplets, with the increase in interfacial viscous hysteresis resistance surpassing the increase in driving force. As shown in Figure 3d, guided by the asymmetric edge of an 808 nm laser, droplets of different volumes can accurately follow the light source to achieve continuous and stable directional movement, confirming the efficiency and broad applicability of the photothermal super-slippery surface in non-contact droplet manipulation.

### 3.4. Multimodal Manipulation of Light Controlled Droplets

**Figure 4:**
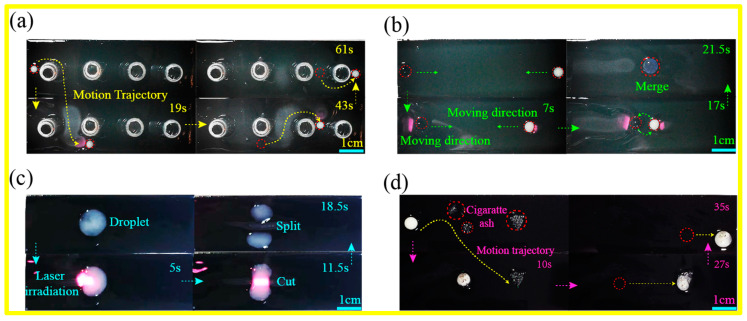
Multimodal manipulation of light controlled droplets: motion, coalescence, fragmentation, and particle capture: (**a**) Controllable obstacle avoidance trajectory motion of droplets. (**b**) Controllable merging of two droplets. (**c**) Laser induced droplet splitting and cutting. (**d**) Droplet mediated surface self-cleaning and pollutant removal.

To evaluate the surface’s ability to manipulate droplets in multiple modes, dynamic droplet propulsion experiments were conducted using an 808 nm, 0.5 W near-infrared laser under ambient conditions. A preset “U-shaped” laser trajectory enables a 5 μL droplet to navigate precisely around obstacles under continuous asymmetric laser irradiation (Figure 4a). The droplet followed the designated at an average speed of 1.63 mm/s, without significant path deviation or interfacial pinning occurring throughout the entire dynamic sliding process. As shown in Figure 4b, by applying photothermal excitation to two droplets (each 10 μL, initial distance of 62 mm) simultaneously, they can be controlled to move towards each other and eventually merge into a larger droplet. Figure 4c shows the laser-induced droplet splitting and cutting process. Focusing the laser on the geometric center of the droplet causes a sharp decrease in surface tension due to local temperature rise, triggering centrifugal Marangoni convection. Under the continuous action of photothermal excitation, the droplet exhibits a “necking” characteristic at 11.5 s, and overcomes the cohesive force at 18.5 s, splitting into two independent droplets. Figure 4d demonstrates the transport of a single droplet at 2.05 mm/s via laser-induced sweeping motion across a surface distributed with cigarette ash particles. When the droplet moves to the location of the soot, the soot particles are rapidly engulfed and encapsulated within the droplet, ultimately restoring it to its initial clean state.

### 3.5. Surface Durability

**Figure 5:**
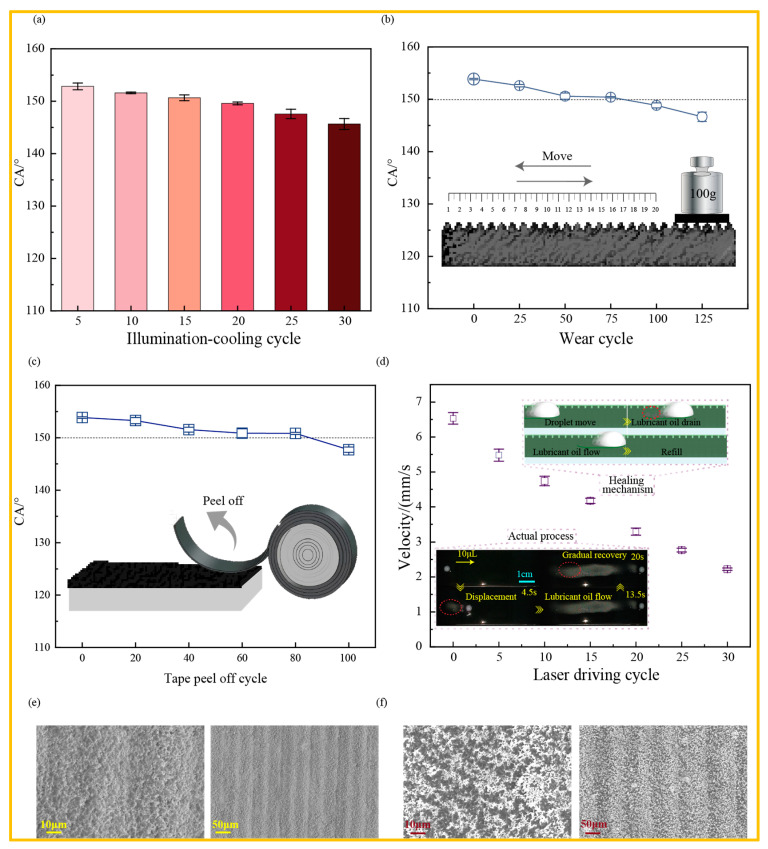
Durability evaluation of a photothermal-responsive surface: (**a**) Cyclic stability of surface photothermal performance. (**b**) Sandpaper abrasion test. (**c**) Adhesive tape peel off test. (**d**) Repeatability of droplet-directed transport. (**e**) SEM morphology after abrasion. (**f**) SEM morphology after adhesive tape peel.

To investigate the durability of the photothermal surface, a surface with a CB content of 3% was selected for durability testing. As shown in Figure 5a, after subjecting the surface to multiple cycles of illumination and cooling, the initial CA of the droplet was 153.86° ± 0.14° and remained stable throughout, with no significant decrease, confirming its excellent resistance to thermal fatigue and photochemical stability. As shown in Figure 5b, the CA of the coating decreased only slightly with increasing abrasion cycles. After 75 abrasion cycles, the CA remained stable at over 150°, indicating that the coating possesses good wear resistance. As shown in Figure 5c, after repeated adhesion and peeling of adhesive tape, the surface CA did not degrade significantly, further demonstrating the coating’s excellent adhesion properties. As shown in the SEM images in Figure 5e,f, even after undergoing high-intensity sandpaper abrasion and repeated tape peeling, the surface’s unique micro-nano scale composite structure remained intact, with no large-scale collapse or coating peeling. This is primarily attributed to the following two factors: first, PDMS, acting as the binder phase, possesses excellent adhesion, which enhances the interfacial bonding strength between the coating and the substrate, effectively suppressing delamination during friction and ensuring the coating’s uniformity and wear resistance; second, the prolonged mechanical stirring process ensures the uniform distribution of micro-nano scale particles on the surface. Even after mechanical wear, the coating retains its micro-nano scale roughness, ensuring the persistence of the superhydrophobic state and demonstrating exceptional mechanical stability. This robust and dense microscopic porous network acts as a “micro-reservoir,” firmly trapping the lubricating oil during droplet movement.

Based on this enduring oil-retention capability, the dynamic, repetitive propulsion of droplets on the surface exhibits extremely high reproducibility. As shown in Figure 5d, in continuous, multiple light-controlled directed transport tests conducted on 10 μL droplets, the droplets were able to maintain smooth sliding motion even after multiple cycles of photothermal propulsion. After 15 repeated drives, the droplet’s movement speed decreased from 6.53 mm/s to 4.17 mm/s; although the speed slowed, it remained superior to the 3.38 mm/s movement speed observed on a surface not treated by laser ablation. This is primarily attributed to the dynamic self-repair mechanism of the lubrication layer: when the heated droplet displaces or consumes the lubricant at the bottom during sliding, the interconnected microscopic channels created by laser ablation allow reserve lubricant “stored” in the surrounding microstructure to rapidly and spontaneously flow back to the depleted areas, refilling the gaps along the droplet’s trajectory. This dynamic closed-loop mechanism of “displacement–reflow–refilling” significantly delays the complete loss of the lubrication layer, ensuring the long-term reliability of the light-controlled droplet’s sustained motion.

## 4. Conclusions

We developed a robust photothermal surface with multi-modal droplet manipulation capability, exceptional mechanical durability, and superior dynamic fluidic control. The PDMS-CB composite coating, fabricated via a combination of spin-coating and laser ablation. This study demonstrates: (1) flexible multi-mode manipulation capabilities, enabling high-speed droplet transport (up to 6.53 mm/s for 10 μL droplets) as well as complex maneuvers including obstacle avoidance, merging, and splitting; and (2) exceptional durability, maintaining stable superhydrophobicity even after repeated cycles of illumination-cooling, abrasion, and tape peeling. Notably, thanks to the dynamic self-healing mechanism of the lubricating layer, the droplet sliding speed remained at 4.17 mm/s after 15 consecutive drives, higher than the 3.38 mm/s observed on photothermal surfaces without laser ablation, demonstrating superior durability. This platform is highly suitable for non-contact microfluidic and lab chip systems. However, its limitations include potential droplet evaporation under prolonged laser irradiation and the requirement for an external light source. Future research will focus on minimizing droplet evaporation and integrating sensors with real-time feedback control systems to enable real-time detection and adaptive regulation of droplet trajectories.

## Data Availability

The original contributions presented in this study are included in the article. Further inquiries can be directed to the corresponding authors.
